# Combination of oncolytic adenovirus targeting SATB1 and docetaxel for the treatment of castration-resistant prostate cancer

**DOI:** 10.7150/jca.46868

**Published:** 2021-01-25

**Authors:** Lijun Mao, Haiyuan Yu, Sai Ma, Ziyang Xu, Fukun Wei, Chunhua Yang, Junnian Zheng

**Affiliations:** 1 Department of Urinary Surgery, the Affiliated Hospital of Xuzhou Medical University, Xuzhou, 221000, China.; 2 Jiangsu Key Laboratory of Biological Cancer Therapy, Xuzhou Medical College, Xuzhou 221002, China.

**Keywords:** SATB1, docetaxel, oncolytic adenovirus, prostate cancer

## Abstract

**Background:** Oncolytic viral therapy is a new strategy for tumor eradication which combines the advantages of viral therapy and gene therapy. Silencing SATB1 exhibits strong inhibitory effect on the growth of prostate cancer. Docetaxel (DTX) is the gold standard for chemotherapy of prostate cancer, but its side effects decrease the life quality of patients. Therefore, it is urgent to develop combination therapy to increase its anti-tumor effect.

**Methods:** Oncolytic adenovirus targeting SATB1 was constructed and named ZD55-SATB1. Human prostatic cancer cells DU145 and PC-3 and human prostatic stromal cells WPMY-1 were treated with ZD55-SATB1 or/and DTX. *In vitro* cell proliferation, migration, invasion, apoptosis were detected. In addition, PC-3 cells were injected subcutaneously into nude mice, which were treated with ZD55-SATB1 or/and DTX. Tumor growth was monitored *in vivo*.

**Results:** ZD55-SATB1 combined with DTX inhibited proliferation, migration and invasion of DU145 and PC-3 cells, while promoted apoptosis of DU145 and PC-3 cells more efficiently than monotherapy. ZD55-SATB1 had no cytotoxicity on WPMY-1 cells. In animal models, the combined treatment of ZD55-SATB1+DTX and endocrine therapy effectively inhibited the growth of xenograft tumor, accompanied by increased expression of caspase-3 and caspase-8, and decrease expression of Bcl-2 and angiogenesis marker CD31 compared to other treatment groups.

**Conclusion:** The combination of oncolytic adenovirus ZD55-SATB1 and chemotherapy provides a novel approach to effective therapy of prostate cancer.

## Introduction

The incidence of prostate cancer has raised up to the second of malignant tumor in men, and its mortality rate is the fifth highest worldwide 1. The diagnostic rate of prostate cancer has increased recently due to the screening of prostate specific antigen (PSA). The optimal treatment for late stage prostate cancer is endocrine therapy. Many prostate cancer patients are initially sensitive to endocrine therapy and experience temporary tumor regression, but nearly all patients develop castration-resistant prostate cancer (CRPC) accompanied by bone metastasis or distant metastasis 2. Docetaxel (DTX) is one of the effective chemotherapy drugs for CRPC 3. Prostate tumor volume was reduced by an average of one third after DTX treatment but tumor remission is reported due to drug resistance to DTX 4.

SATB1 (special AT-rich sequence binding-protein 1) is a global chromatin organizer that could regulate gene expression and chromatin structure 5. SATB1 is abnormally overexpressed in a variety of cancers and is proposed as an oncogene to promote tumor growth and invasion 6-10. Therefore, SATB1 became a target for cancer therapy.

Gene viral therapy of cancer is a new strategy for tumor eradication which combines the advantages of viral therapy and gene therapy. Oncolytic adenovirus can self-replicate in tumor cells, lyse tumor cells and infect neighboring tumor cells. Oncolytic adenovirus has high anti-tumor efficacy and safety 11. In particular, E1B55-kDa gene defective oncolytic adenoviruses (Ad-ZD55) not only efficiently infects and replicates in tumor cells, but also amplifies IL-24 gene to enhance the expression and function of IL-24 in tumor microenvironment without affecting adjacent normal cells 12.

In this study, we constructed oncolytic adenovirus carrying SATB1 named as Ad-ZD55-SATB1 (ZD55-SATB1) and combined it with chemotherapeutic drug DTX to explore the effects on proliferation, invasion, and apoptosis of prostate cancer cells *in vitro* and *in vivo*.

## Materials and Methods

### Cell culture

Human prostatic cancer cell line DU145 and PC-3, and human prostatic stromal cell line WPMY-1 were obtained from Chinese Academy of Sciences and cultured in RPMI-1640 or DMEM medium with 10% fetal bovine serum (Gibco, USA) at 37°C in a humidified incubator with 5% CO_2_. DTX was obtained from Jiangsu Hengrui Medicine Co, Ltd.

### Recombinant adenoviruses

ZD55-SATB1 and ZD55-EGFP were provided by Jiangsu Key Laboratory of Biological Cancer Therapy, the viruses were purified using Adenovirus Purification Mini Kit (Biomiga, Inc. CA, USA) and stored at 4°C. The virus titer was measured using QuickTiterTM Kit (Cell Biolabs, CA, USA).

### Cell viability assay

Cell proliferation was tested by Cell Counting kit‑8 (CCK‑8) assay. DU145, PC-3, and WPMY-1 cells were seeded in 96-well plates and cultured for 24 h. Then cells were treated with DTX (1 ng/mL) or/and ZD55-SATB1 or ZD55-EGFP at 10 multiplicity of infection (MOI) for 24, 48, 72 and 96 h. After incubation with CCK-8 solution the absorbance at 450 nm was measured.

### Cell invasion and migration assay

PC-3 and DU145 cells were seeded in the top of matrigel-coated and uncoated (BD Biosciences; Bedford, MA, USA) transwell chambers (Corning, Corning, NY, USA), and the lower chamber was filled with 20% FBS in RPMI 1640 medium. After incubation for 24 h, cells that had invaded or migrated into the lower chamber were fixed in 4% paraformaldehyde and stained with crystal violet. Stained cells were counted under microscopy.

### Apoptosis assay

PC-3 and DU145 cells were harvested after 48 h of treatment and stained using Annexin V-FITC/propidium iodide double staining kit (Keygen Biotech; Nanjing, China). Stained cells were immediately subjected to flow cytometry.

### Western blot analysis

Cells were lysed in lysis buffer with protease inhibitor, and equal amounts of lysates were separated by SDS-PAGE and transferred onto the membranes, which were incubated with antibodies for SATB1 (Abcam, UK, #ab109122), MMP-2 (Abcam, UK, #ab92536), Vimentin (Abcam, UK, #ab137321), E-Cadherin (Abcam, UK, # ab201499), Bcl-2 (Abcam, UK, #ab194583), caspase-3 (Abcam, UK, #ab197202), caspase-8 (Abcam, UK, #ab108333), GAPDH (Abcam, UK, #ab37168). The blots were washed, incubated with secondary antibodies (Abcam, UK), and developed using enhanced chemiluminescence (ECL) system (Pierce, Rockford, IL, USA).

### Subcutaneous tumor model in mice

Male BALB/c nude mice (5 weeks old) were purchased from Vital River Laboratory (Beijing, China) and quarantined for a week. Experimental procedures were approved by Committee of Care and Use of Laboratory Animals of Xuzhou Medical University. 1×10^6^ PC-3 cells were injected subcutaneously into each mouse, and the mice were randomly divided into 8 groups (n=8): (1) control group (Con): received intraperitoneal injection of PBS; (2) endocrine therapy group (ET): received surgical removal of the bilateral testicles; (3) drug alone group (DTX): received intraperitoneal injection of DTX (10 mg/kg) once a week; (4) adenovirus treatment group (ZD55-SATB1): received intratumoral injection of ZD55-SATB1 (1×10^9^pfu) every three days; (5) ET + ZD55-SATB1 group: received surgical castration and intratumoral injection of ZD55-SATB1; (6) ET + DTX: received surgical castration and intraperitoneal injection of DTX (10 mg/kg) once a week; (7) ZD55-SATB1 + DTX group: received intratumoral injection of ZD55-SATB1 and intraperitoneal injection of DTX; (8) ET + ZD55-SATB1 + DTX group: received surgical castration, intratumoral injection of ZD55-SATB1 and intraperitoneal injection of DTX. Tumor volume was measured every three days using the formula: V (mm^3^) =the length× width^2^×0.5. On the 28th day after injection the mice were sacrificed and xenograft tumors were dissected. The tumors were stained with hematoxylin-eosin following standard protocols, and immunohistochemical staining was performed using the antibodies for caspase-8, caspase-3, CD31 and Bcl-2 (Abcam, UK) and DBA kit (ZSGB-Bio, Beijing, China).

### Statistical analysis

Data are shown as mean± standard deviation (SD). Comparison was performed by T-test for two groups and one-way ANOVA for multiple groups. P<0.05 was considered significant.

## Results

### Combined treatment effectively and specially inhibited the viability of prostate cancer cells

**Figure 1:**
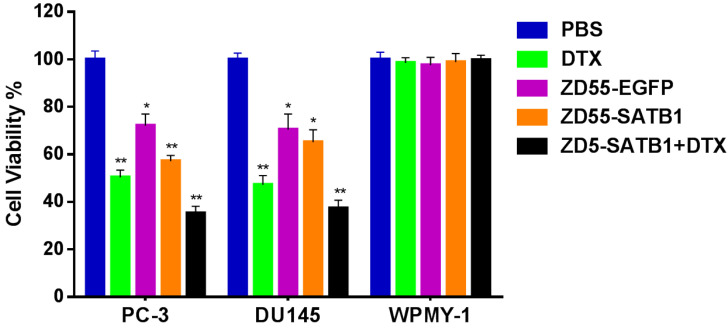
The viability of prostate cells in different treatment groups. *P<0.05, **P<0.01, versus PBS group.

We chose three cell lines: PC-3 human prostate cancer cells with high metastatic potential; DU145 human prostate cancer cells with moderate metastatic potential, and WPMY-1 human prostatic stromal cells as control. Cells were divided into five groups: treated with PBS as normal control; treated with DTX as chemotherapy group; treated with ZD55-EGFP as oncolytic therapy group; treated with ZD55-SATB1 as oncolytic therapy and SATB1 targeted therapy group; treated with ZD55-SATB1+ DTX as oncolytic therapy and SATB1 targeted therapy in combination with chemotherapy. CCK-8 assay showed that the viability of PC-3 cells after 48 h in the ZD55-SATB1 (10 MOI) plus DTX (1 ng/mL) group (35.16±2.17%) was significantly lower than ZD55-SATB1 (10 MOI) group (57.13±2.02%); ZD55-EGFP (10 MOI) group (72.25±5.49%) and DTX (1 ng/mL) group (50.16±3.27%). The viability of DU145 cells after 48 h in the ZD55-SATB1 (10 MOI) plus DTX (1 ng/mL) group (37.27±3.62%) was significantly lower than ZD55-SATB1 (10 MOI) group (65.16±5.49%); ZD55-EGFP and (10 MOI) group (70.45±6.71%); DTX (1 ng/mL) group (47.19±3.56%). Finally, ZD55-SATB1 combined with DTX had little or no obvious effects on WPMY-1 cells (Figure 1).

### Combined treatment effectively inhibited the invasion and migration of prostate cancer cells

**Figure 2:**
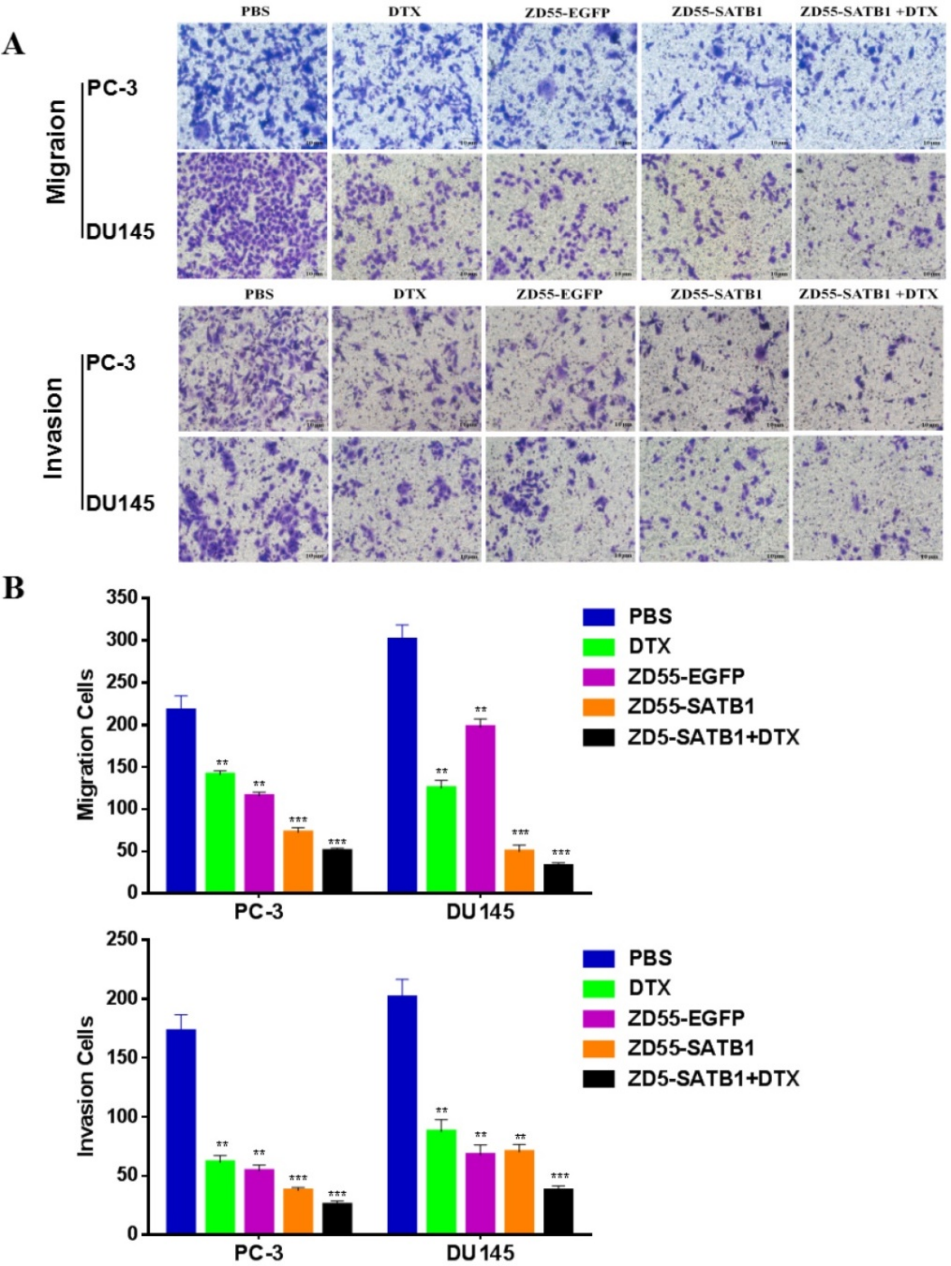
The invasion and migration of prostate cancer cells in different treatment groups. A. Images of transwell assays with PC-3 and DU145 cells (×200). B. Quantification of invaded and migrated cells in transwell assays. Data are presented as the mean±SD (n=3). **P<0.01, ***P<0.001, versus PBS group.

Transwell assay showed that the numbers of invaded PC-3 cells in PBS, DTX, ZD55-EGFP, ZD55-SATB1 and DTX+ZD55-SATB1 groups were 172.2 ±14.5, 61.1±4.8, 54.4±4.4, 37.1±2.2, 25.7±3.6, respectively; the numbers of invaded DU145 cells in PBS, DTX, ZD55-EGFP, ZD55-SATB1 and DTX+ZD55-SATB1 groups were 201.7 ±15.4, 87.7±9.9, 67.6±8.5, 70.6±6.7, 37.5±4.1, respectively; the numbers of migrated PC-3 cells in PBS, DTX, ZD55-EGFP, ZD55-SATB1 and DTX+ZD55-SATB1 groups were 217.1 ±14.2, 141.7±4.4, 115.3±4.6, 72.7±6.2, 50.2±3.1, respectively; the numbers of migrated DU145 cells in PBS, DTX, ZD55-EGFP, ZD55-SATB1 and DTX+ ZD55-SATB1 groups were 301.2±17.3, 125.5±9.6, 197.2±8.7, 49.7±6.6, 32.4±4.7, respectively (Figure 2A,B). These results indicate that ZD55-SATB1+DTX exhibit much greater potential in suppressing migratory and invasive ability of prostate cancer cells compared with monotherapy group.

### Combined treatment effectively induced the apoptosis of prostate cancer cells

**Figure 3:**
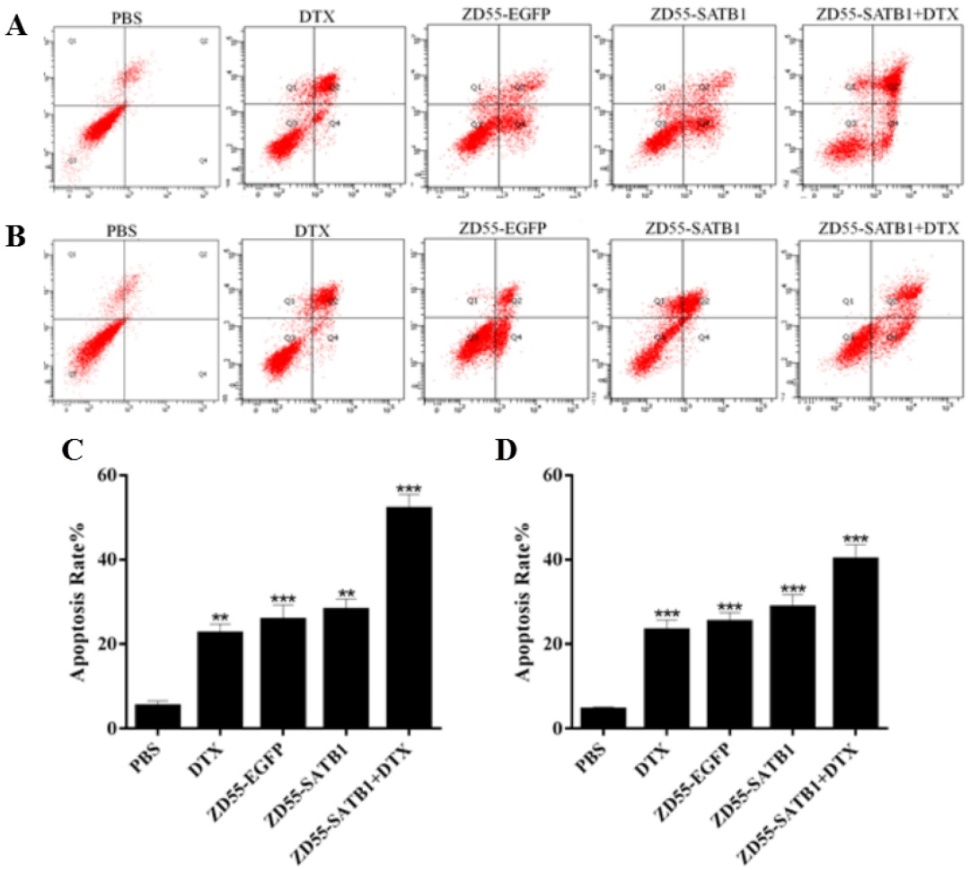
The apoptosis of prostate cancer cells in different treatment groups. The apoptosis of PC-3 (A) and DU145 (B) cells after 48 h treatment was detected by flow cytometric analysis. Quantitative analysis of the percentage of apoptosis of PC-3 (C) and DU145 (D) cells. Data are expressed as mean± SD of three separate experiments (**P<0.01, ***P<0.001 versus PBS group).

Flow cytometry based on Annexin-V-FITC/propidium iodide double staining showed that the percentage of apoptosis for PC-3 cells treated with ZD55-SATB1+DTX (52.19±3.46%) significantly increased compared to ZD55-SATB1 (28.17±2.31%) (P<0.01), ZD55-EGFP (25.86±3.53%) (P<0.01), DTX (22.58±2.24%) (P<0.01), while the percentage of apoptosis in the control group was significantly lower (5.32±1.28%) (Figure 3A,C). Similarly, combined treatment of ZD55-SATB1 and DTX significantly elevated the percentage of apoptosis for DU145 cells compared to either ZD55-SATB1 (28.79±3.04%) or DTX (23.33±2.47%) (Figure 3B,D). These results demonstrate that the combined treatment of ZD55-SATB1 with DTX efficiently induced the apoptosis of prostate cancer cells compared with monotherapy.

### Combined treatment effectively inhibited epithelial-mesenchymal transition of prostate cancer cells

**Figure 4:**
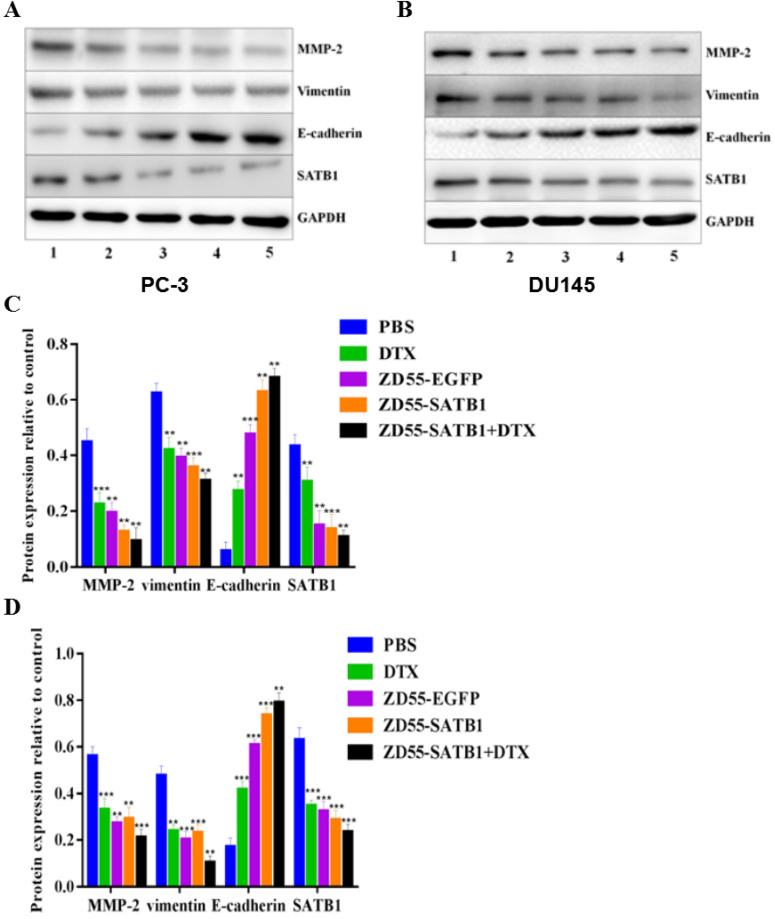
The expression of EMT related proteins in different treatment groups. Western blot analysis of MMP-2, Vimentin, E-cadherin, SATB1 in PC-3 (A) and DU145 (B) cells. GAPDH was loading control. 1. PBS; 2. DTX; 3. ZD55-EGFP; 4. ZD55-SATB1; 5. ZD55-SATB1+DTX. Densitometry analysis of protein levels in PC-3 cells (C) and DU145 cells (D). Experiments were performed independently for three times. (**P<0.01, ***P<0.001 versus PBS group).

Epithelial mesenchymal transition (EMT) is associated with cancer invasiveness and migration. To examine whether the prostate cancer cell migration and invasion were associated with the EMT, we detected the expression of EMT related proteins, including MMP-2, vimentin and E-cadherin in PC-3 and DU145 cells. Combined ZD55-SATB1 and DTX significantly down-regulated the expression level of MMP-2, Vimentin and SATB1 and up-regulated the expressions of E-cadherin compared to ZD55-SATB1 or DTX monotherapy group (Figure 4, all P<0.05). These data suggest that combination treatment with ZD55-SATB1 and DTX inhibits prostate cancer metastasis and invasion via the inhibition of EMT.

### Combined treatment effectively inhibited the growth of tumor xenograft in nude mice

**Figure 5:**
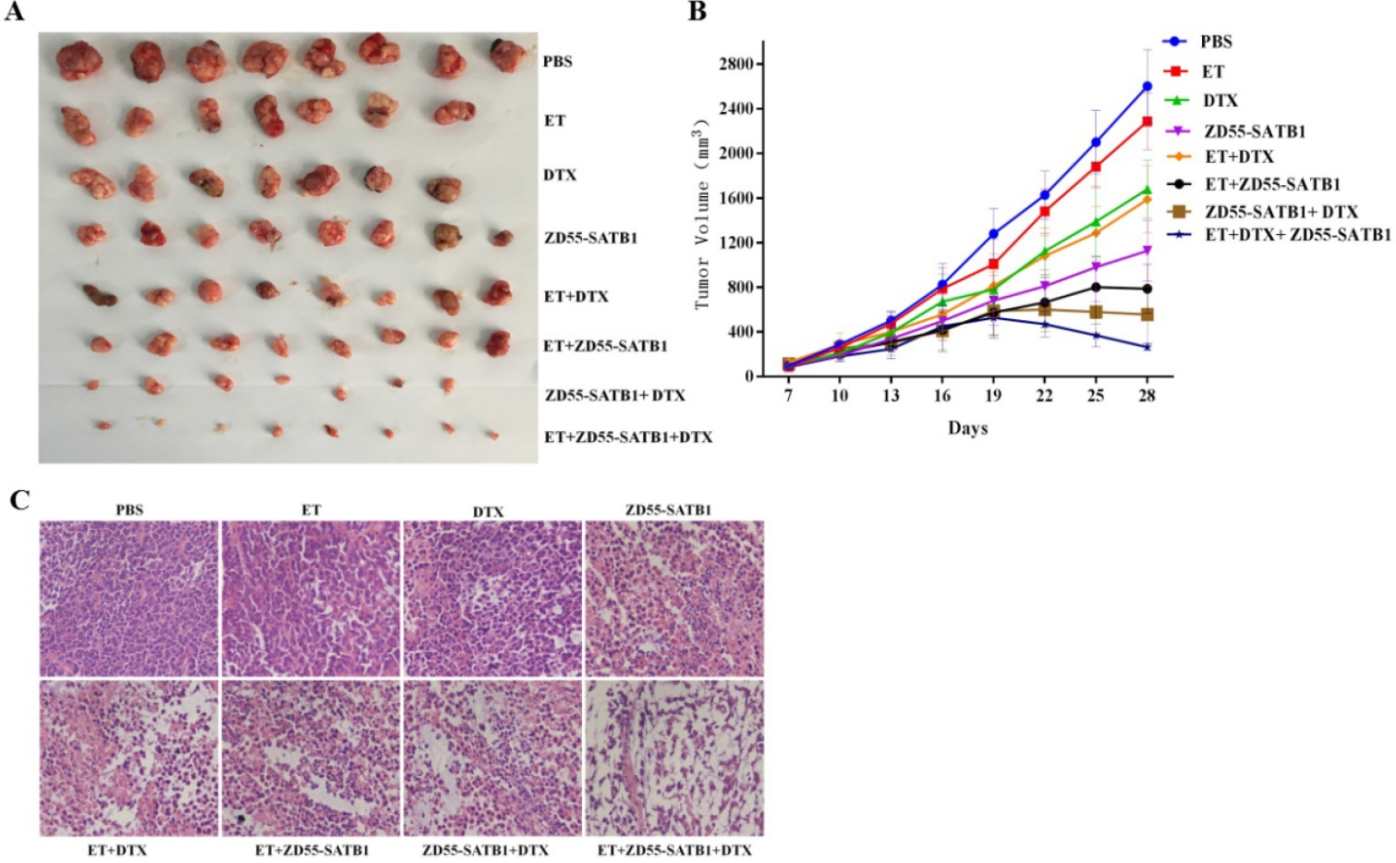
*In vivo* tumor growth in different treatment groups. A. PC-3 prostate tumor xenografts were harvested from each group. B. Tumor volume was measured at 3-day intervals and tumor growth curves were drawn to show growth of tumors of each group. C. H&E staining of xenografts collected from different groups of mice (×400).

Based on the above *in vitro* data, we further evaluated the treatment effects of ZD55-SATB1 combined DTX on prostate cancer growth *in vivo*. PC-3 cells were injected subcutaneously into nude mice and 8 groups received different treatments a week later. The tumors were harvested 28 days later (Figure 5A). Tumor growth curve showed that ZD55-SATB1 combined with DTX and ET led to the slowest growth of xenograft tumors (Figure 5B). Hematoxylin-eosin staining showed reduced tumor in ZD55-SATB1 combined with DTX and ET group (Figure 5C).

### Combined treatment effectively induced the apoptosis and inhibited the angiogenesis of tumor xenograft in nude mice

**Figure 6:**
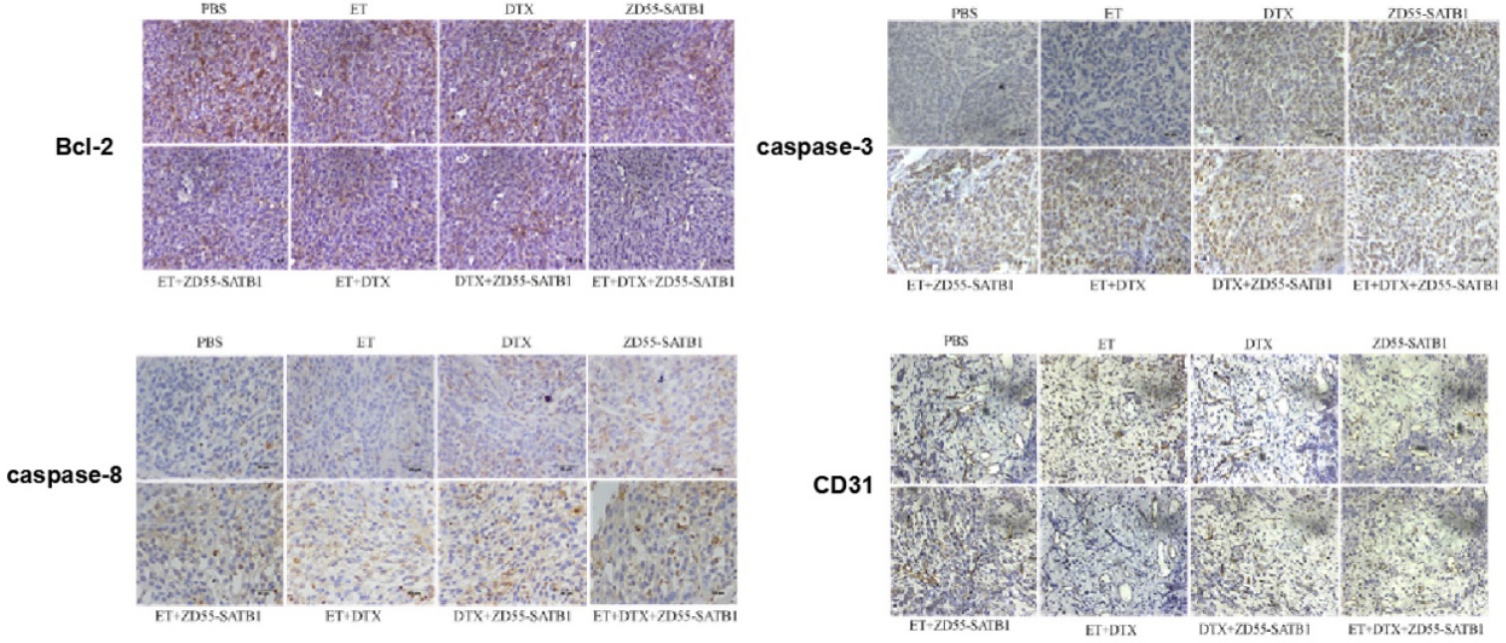
Immunohistochemical staining of caspase-3, caspase-8, Bcl-2 and CD31 in PC-3 prostate tumor xenografts in nude mice. (×200).

Immunohistochemical staining showed that ZD55-SATB1, DTX or ET alone increased the expression of caspase-3 and caspase-8 and decreased Bcl-2 expression. However, ZD55-SATB1+DTX+ET led to greater increase in the expression of caspase-3 and caspase-8 and greater decrease in Bcl-2 expression compared to other treatment groups. Moreover, ZD55-SATB1+DTX+ET led to greater decrease in the expression of angiogenesis marker CD31 compared to other treatment groups (Figure 6).

## Discussion

Most prostate cancers are androgen dependent and androgen deprivation therapy (ADT) becomes the preferred treatment for advanced prostate cancer. Although ADT is efficient in early treatment, most patients developed CRPC about 1-3 years after ADT 13. DTX based chemotherapy is recommended as a first-line treatment for prostate cancer. However, side effects of DTX such as myelosuppression, fluid retention, anaphylaxis, hypotension, vomiting or diarrhea affect the life quality of patients severely 14.

In this study we combined DTX with oncolytic adenovirus carrying SATB1 (ZD55-SATB1) to enhance the efficacy of DTX and reduce the dose and possible side effects of DTX. *In vitro* experiments showed that combined treatment (ZD55-SATB1 plus DTX) had the highest efficacy to inhibit the viability, invasion and migration of prostate cancer cells while had no significant effect on the viability of normal prostate cells. In addition, combined treatment had the highest efficacy to induce the apoptosis of prostate cancer cells.

EMT is a crucial process in cancer invasion and metastasis 15. E-cadherin and vimentin are both EMT related proteins, and MMP-2 plays an important role in tumor invasion and metastasis 16,17. Thus we performed Western blot analysis to detect the expression of MMP-2, vimentin and E-cadherin in PC-3 and DU145 cells. In cells treated with ZD55-SATB1 plus DTX, the expression levels of MMP-2, vimentin and SATB1 were significantly downregulated while the expressions of E-cadherin was significantly upregulated compared to other groups. Collectively, these results indicate that ZD55-SATB1 combined with DTX may inhibit prostate cancer growth by inducing apoptosis and inhibit prostate cancer invasion and metastasis by reversing EMT.

To further validate the anti-tumor efficacy of combined treatment, we employed nude mouse model with xenograft of prostate cancer cells. Monitoring of tumor growth in nude mice showed that tumor size was the smallest in ZD55-SATB1 combined with DTX and ET group. Immunohistochemical staining showed that apoptosis related proteins exhibited the most significant changes in expression levels in ZD55-SATB1+DTX+ET group compared to other combined treatment group and monotherapy group. These results are consistent with *in vitro* data that combined treatment induced the apoptosis of prostate cancer cells.

CD31 (also named platelet endothelial cell adhesion molecule-1) is expressed in blood vessels and lymphatic endothelial cells, and participates in angiogenesis and tumor metastasis 18. Therefore, we detected CD31 expression in tumor tissues of nude mice by immunohistochemical staining. CD31 level significantly decreased in ZD55-SATB1+DTX+ET group compared to other combined treatment group and monotherapy group. These data complement the *in vitro* data that ZD55-SATB1+DTX could inhibit the invasion of prostate cancer cells.

In conclusion, the combination of ZD55-SATB1 and DTX showed synergistic effects to inhibit prostate cancer cell proliferation, invasion and migration, while induce prostate cancer cell apoptosis. The anti-metastasis mechanism of combined treatment is related to the inhibition of EMT. The combination of ZD55-SATB1 and DTX with endocrine therapy may provide a new approach to the treatment of prostate cancer.
